# K_2_Ho(PO_4_)(WO_4_)

**DOI:** 10.1107/S160053680803287X

**Published:** 2008-10-18

**Authors:** Katherina V. Terebilenko, Igor V. Zatovsky, Vyacheslav N. Baumer, Nikolay S. Slobodyanik, Oleg V. Shishkin

**Affiliations:** a Department of Inorganic Chemistry, Taras Shevchenko National University, 64 Volodymyrska Street, 01033 Kyiv, Ukraine; b STC ‘Institute for Single Crystals’, NAS of Ukraine, 60 Lenin Avenue, 61001 Kharkiv, Ukraine

## Abstract

A new compound, dipotassium holmium(III) phosphate(V) tungstate(VI), K_2_Ho(PO_4_)(WO_4_), has been obtained during investigation of the K_2_O–P_2_O_5_–WO_3_–HoF_3_ phase system using the flux technique. The compound is isotypic with K_2_Bi(PO_4_)(WO_4_). Its framework structure consists of flat _∞_^2^[HoPO_4_] layers parallel to (100) that are made up of _∞_^1^[HoO_8_] zigzag chains inter­linked *via* slightly distorted PO_4_ tetra­hedra. WO_4_ tetra­hedra are attached above and below these layers, leaving space for the K^+^ counter-cations. The HoO_8_, PO_4_ and WO_4_ units exhibit 2 symmetry.

## Related literature

For related structures, see: Ben Amara & Dabbabi (1987 ▶); Marsh (1987 ▶); Zatovsky, Terebilenko, Slobodyanik & Baumer (2006 ▶); Zatovsky, Terebilenko, Slobodyanik, Baumer & Shishkin (2006 ▶).

## Experimental

### 

#### Crystal data

K_2_Ho(PO_4_)(WO_4_)
*M* *_r_* = 585.95
Orthorhombic, 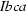

*a* = 6.8820 (10) Å
*b* = 12.1485 (18) Å
*c* = 19.695 (3) Å
*V* = 1646.6 (4) Å^3^
*Z* = 8
Mo *K*α radiation
μ = 24.72 mm^−1^
*T* = 293 (2) K
0.10 × 0.09 × 0.07 mm

#### Data collection

Oxford Diffraction Xcalibur-3 CCD diffractometer
Absorption correction: multi-scan based on the method by Blessing (1995 ▶) *T*_min_ = 0.102, *T*_max_ = 0.177
8608 measured reflections
1561 independent reflections
1257 reflections with *I* > 2σ(*I*)
*R*_int_ = 0.055

#### Refinement

*R*[*F* ^2^ > 2σ(*F* ^2^)] = 0.029
*wR*(*F* ^2^) = 0.071
*S* = 1.15
1561 reflections
62 parameters
Δρ_max_ = 1.90 e Å^−3^
Δρ_min_ = −1.73 e Å^−3^

### 

Data collection: *CrysAlis CCD* (Oxford Diffraction, 2005 ▶); cell refinement: *CrysAlis RED* (Oxford Diffraction, 2005 ▶); data reduction: *CrysAlis RED*; program(s) used to solve structure: *SHELXS97* (Sheldrick, 2008 ▶); program(s) used to refine structure: *SHELXL97* (Sheldrick, 2008 ▶); molecular graphics: *DIAMOND* (Brandenburg, 2006 ▶); software used to prepare material for publication: *WinGX* (Farrugia, 1999 ▶).

## Supplementary Material

Crystal structure: contains datablocks global, I. DOI: 10.1107/S160053680803287X/wm2196sup1.cif

Structure factors: contains datablocks I. DOI: 10.1107/S160053680803287X/wm2196Isup2.hkl

Additional supplementary materials:  crystallographic information; 3D view; checkCIF report

## Figures and Tables

**Table 1:** Selected bond lengths (Å)

W1—O2	1.749 (4)
W1—O1	1.788 (3)
Ho1—O4^i^	2.274 (3)
Ho1—O1	2.342 (3)
Ho1—O3^ii^	2.401 (3)
Ho1—O4	2.428 (3)
P1—O3	1.525 (3)
P1—O4	1.552 (3)

Symmetry codes: (i) 
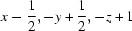
; (ii) 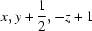.

**Fig. 1.**
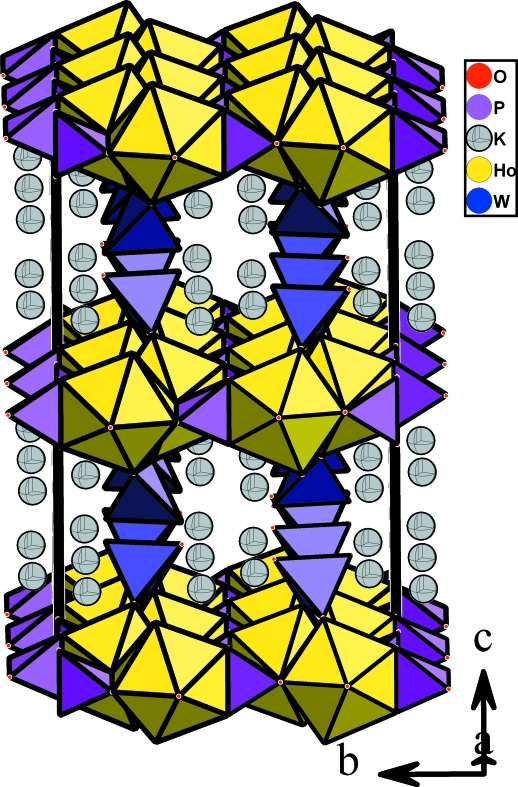
The layered structure of K2Ho(PO4)(WO4), leaving space where the K+ ions are located.

**Fig. 2.**
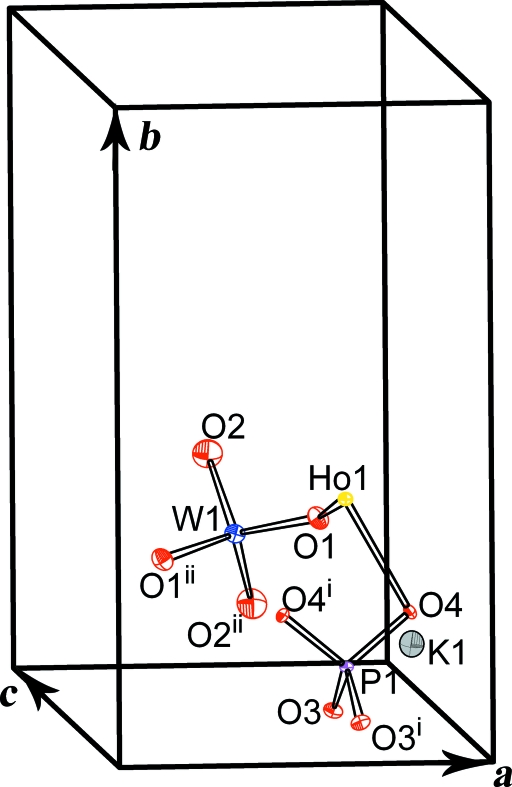
View of K2Ho(PO4)(WO4) with displacement ellipsoids drawn at the 50% probability level. [Symmetry codes: (i) 1,5-x; y; 1-z; (ii) 1-x; 0.5-y; z].

## supplementary crystallographic information

### Comment

The co-existence of different anionic units in crystal structures represents an interesting field of investigation. One of the first structural examples of a combination of PO_4_ with MoO_4_/WO_4_ tetrahedra, *viz.* Na_2_Y(MoO_4_)(PO_4_), was reported to be monoclinic with space group *C*2/*c* (Ben Amara & Dabbabi, 1987). Later this structure was reinvestigated and described as orthorhombic, space group *Ibca* (Marsh, 1987). Recently, the compounds K_2_Bi(PO_4_)(MO_4_) (*M*=Mo, W) with isotypic structures were obtained by application of the flux method (Zatovsky, Terebilenko, Slobodyanik & Baumer, 2006; Zatovsky, Terebilenko, Slobodyanik, Baumer & Shishkin, 2006). Herein, we report the flux synthesis and crystal structure of a new member of the *A*_2_*B*(PO_4_)(*A*O_4_) (*A* = Na, K; *B* = lanthanide, Y, Bi; *M* = Mo, W) family.

One of the characteristic features of this structure type is the "segregation" of slightly distorted PO_4_ and WO_4_ tetrahedra into adjacent layers (Fig. 1). The first layer with composition ^2^_∞_[HoPO_4_] contains ^1^_∞_[HoO_8_] zigzag chains (Fig. 2). The connection between neighboring chains is achieved *via* PO_4_ tetrahedra. On the top and on the bottom of the ^2^_∞_[HoPO_4_] layer, WO_4_ tetrahedra are attached. All [HoO_8_], PO_4_ and WO_4_ units exhibit 2 symmetry with bond lengths in the typical ranges (Table 1). The K^+^ cations are situated in the resulting interlayer space and are surrounded by 8 oxygen atoms with K—O bond lengths ranging from 2.683 (4) Å to 3.133 (4) Å.

### Experimental

Single crystals of the title compound were grown from a multicomponent high-temperature solution. A mixture of 4.645 g K_2_W_2_O_7_, 0.865 g KPO_3_, and 1.150 g K_4_P_2_O_7_ was heated in a platinum crucible up to 1173 K which is above the melting temperature. Then 0.200 g of HoF_3_ were added to this melt under stirring. The final mixture was held at this temperature for 1 h and cooled down to room temperature with a rate of 30 Kh^-1^. The solidified melt was leached out with warm water to dissolve the superfluous flux. The final product consisted of beige needle-like crystals with a maximum length of up to 5 mm.

### Refinement

The highest peak and the deepest hole of the final Fourier map are located 0.58 Å from atom W1 and 1.11 Å from the same atom, respectively.

### Crystal data

**Table:**

K_2_Ho(PO_4_)(WO_4_)	*F*(000) = 2064
*M**_r_* = 585.95	*D*_x_ = 4.727 Mg m^−^^3^
Orthorhombic, *I**b**c**a*	Mo *K*α radiation, λ = 0.71073 Å
Hall symbol: -I 2b 2c	Cell parameters from 8608 reflections
*a* = 6.882 (1) Å	θ = 3.4–33.0°
*b* = 12.1485 (18) Å	µ = 24.72 mm^−^^1^
*c* = 19.695 (3) Å	*T* = 293 K
*V* = 1646.6 (4) Å^3^	Prism, pale beige
*Z* = 8	0.10 × 0.09 × 0.07 mm

### Data collection

**Table:**

Oxford Diffraction XCalibur-3 CCD diffractometer	1561 independent reflections
Radiation source: fine-focus sealed tube	1257 reflections with *I* > 2σ(*I*)
graphite	*R*_int_ = 0.055
φ and ω scans	θ_max_ = 33.0°, θ_min_ = 3.4°
Absorption correction: multi-scan based on the method by Blessing (1995)	*h* = −10→10
*T*_min_ = 0.102, *T*_max_ = 0.177	*k* = −18→18
8608 measured reflections	*l* = −30→29

### Refinement

**Table:**

Refinement on *F*^2^	0 restraints
Least-squares matrix: full	*w* = 1/[σ^2^(*F*_o_^2^) + (0.0397*P*)^2^ + 3.2575*P*] where *P* = (*F*_o_^2^ + 2*F*_c_^2^)/3
*R*[*F*^2^ > 2σ(*F*^2^)] = 0.029	(Δ/σ)_max_ < 0.001
*wR*(*F*^2^) = 0.071	Δρ_max_ = 1.90 e Å^−^^3^
*S* = 1.15	Δρ_min_ = −1.73 e Å^−^^3^
1561 reflections	Extinction correction: SHELXL97 (Sheldrick, 2008), Fc^*^=kFc[1+0.001xFc^2^λ^3^/sin(2θ)]^-1/4^
62 parameters	Extinction coefficient: 0.00010 (2)

### Special details

**Table:**

Geometry. All e.s.d.'s (except the e.s.d. in the dihedral angle between two l.s. planes) are estimated using the full covariance matrix. The cell e.s.d.'s are taken into account individually in the estimation of e.s.d.'s in distances, angles and torsion angles; correlations between e.s.d.'s in cell parameters are only used when they are defined by crystal symmetry. An approximate (isotropic) treatment of cell e.s.d.'s is used for estimating e.s.d.'s involving l.s. planes.

### Fractional atomic coordinates and isotropic or equivalent isotropic displacement parameters (Å^2^)

**Table:**

	*x*	*y*	*z*	*U*_iso_*/*U*_eq_	
W1	0.5	0.25	0.334530 (12)	0.01088 (5)	
Ho1	0.75	0.325113 (17)	0.5	0.00662 (5)	
K1	0.96872 (15)	0.07992 (10)	0.34389 (5)	0.0196 (2)	
P1	0.75	0.07042 (10)	0.5	0.0065 (2)	
O1	0.7088 (4)	0.2796 (3)	0.38536 (17)	0.0146 (7)	
O2	0.4420 (6)	0.3643 (4)	0.2845 (2)	0.0269 (8)	
O3	0.7308 (4)	−0.0047 (2)	0.43834 (16)	0.0102 (6)	
O4	0.9229 (4)	0.1514 (2)	0.49215 (17)	0.0105 (6)	

### Atomic displacement parameters (Å^2^)

**Table:**

	*U*^11^	*U*^22^	*U*^33^	*U*^12^	*U*^13^	*U*^23^
W1	0.01432 (9)	0.01167 (9)	0.00666 (9)	−0.00095 (8)	0	0
Ho1	0.00652 (9)	0.00523 (9)	0.00811 (10)	0	−0.00005 (9)	0
K1	0.0204 (4)	0.0248 (5)	0.0137 (4)	−0.0002 (4)	0.0021 (3)	0.0039 (4)
P1	0.0064 (5)	0.0034 (5)	0.0098 (6)	0	0.0000 (5)	0
O1	0.0154 (14)	0.0171 (14)	0.0113 (14)	−0.0035 (11)	−0.0012 (11)	−0.0031 (11)
O2	0.0308 (17)	0.0296 (19)	0.0203 (17)	−0.0008 (17)	0.0013 (15)	0.0126 (16)
O3	0.0126 (13)	0.0079 (11)	0.0101 (13)	−0.0018 (10)	−0.0002 (11)	−0.0010 (9)
O4	0.0052 (10)	0.0066 (11)	0.0198 (16)	−0.0017 (9)	0.0011 (10)	−0.0016 (11)

### Geometric parameters (Å, °)

**Table:**

W1—O2^i^	1.749 (4)	Ho1—K1^vii^	3.8119 (11)
W1—O2	1.749 (4)	Ho1—K1^vi^	3.8119 (12)
W1—O1	1.788 (3)	K1—O3	2.683 (3)
W1—O1^i^	1.788 (3)	K1—O2^v^	2.689 (4)
W1—K1	3.8352 (12)	K1—O3^x^	2.747 (3)
W1—K1^i^	3.8352 (12)	K1—O1^vi^	2.916 (3)
W1—K1^ii^	4.0181 (13)	K1—O2^xi^	2.934 (5)
W1—K1^iii^	4.0181 (13)	K1—O4	3.063 (3)
W1—K1^iv^	4.0821 (12)	K1—O1	3.122 (4)
W1—K1^v^	4.0821 (12)	K1—O2^i^	3.133 (4)
Ho1—O4^vi^	2.274 (3)	K1—P1	3.4251 (11)
Ho1—O4^vii^	2.274 (3)	K1—Ho1^vi^	3.8119 (11)
Ho1—O1	2.342 (3)	K1—K1^iii^	3.9511 (13)
Ho1—O1^viii^	2.342 (3)	P1—O3	1.525 (3)
Ho1—O3^ix^	2.401 (3)	P1—O3^viii^	1.525 (3)
Ho1—O3^ii^	2.401 (3)	P1—O4	1.552 (3)
Ho1—O4^viii^	2.428 (3)	P1—O4^viii^	1.552 (3)
Ho1—O4	2.428 (3)	P1—Ho1^xii^	2.9802 (13)
Ho1—P1^ix^	2.9802 (13)	P1—K1^viii^	3.4251 (11)
Ho1—P1	3.0941 (13)		
			
O2^i^—W1—O2	111.4 (3)	O3^x^—K1—O4	61.07 (8)
O2^i^—W1—O1	106.94 (17)	O1^vi^—K1—O4	69.23 (9)
O2—W1—O1	109.83 (19)	O2^xi^—K1—O4	130.90 (10)
O2^i^—W1—O1^i^	109.83 (19)	O3—K1—O1	76.52 (9)
O2—W1—O1^i^	106.94 (17)	O2^v^—K1—O1	100.44 (12)
O1—W1—O1^i^	111.9 (2)	O3^x^—K1—O1	117.21 (9)
O4^vi^—Ho1—O4^vii^	165.59 (15)	O1^vi^—K1—O1	84.72 (10)
O4^vi^—Ho1—O1	94.81 (11)	O2^xi^—K1—O1	156.62 (10)
O4^vii^—Ho1—O1	88.59 (11)	O4—K1—O1	58.09 (8)
O4^vi^—Ho1—O1^viii^	88.59 (11)	O3—K1—O2^i^	77.94 (10)
O4^vii^—Ho1—O1^viii^	94.81 (11)	O2^v^—K1—O2^i^	78.50 (9)
O1—Ho1—O1^viii^	152.68 (18)	O3^x^—K1—O2^i^	156.48 (11)
O4^vi^—Ho1—O3^ix^	88.91 (10)	O1^vi^—K1—O2^i^	131.64 (12)
O4^vii^—Ho1—O3^ix^	78.65 (10)	O2^xi^—K1—O2^i^	103.49 (13)
O1—Ho1—O3^ix^	133.18 (11)	O4—K1—O2^i^	101.64 (9)
O1^viii^—Ho1—O3^ix^	73.88 (12)	O1—K1—O2^i^	54.04 (10)
O4^vi^—Ho1—O3^ii^	78.65 (10)	O3—P1—O3^viii^	106.4 (2)
O4^vii^—Ho1—O3^ii^	88.91 (10)	O3—P1—O4	111.52 (16)
O1—Ho1—O3^ii^	73.88 (12)	O3^viii^—P1—O4	113.10 (16)
O1^viii^—Ho1—O3^ii^	133.18 (12)	O3—P1—O4^viii^	113.10 (16)
O3^ix^—Ho1—O3^ii^	61.17 (15)	O3^viii^—P1—O4^viii^	111.52 (16)
O4^vi^—Ho1—O4^viii^	126.77 (7)	O4—P1—O4^viii^	101.3 (2)
O4^vii^—Ho1—O4^viii^	67.63 (12)	W1—O1—Ho1	133.20 (16)
O1—Ho1—O4^viii^	78.27 (12)	W1—O1—K1^vi^	124.93 (16)
O1^viii^—Ho1—O4^viii^	78.03 (12)	Ho1—O1—K1^vi^	92.27 (11)
O3^ix^—Ho1—O4^viii^	133.56 (10)	W1—O1—K1	99.08 (14)
O3^ii^—Ho1—O4^viii^	143.86 (10)	Ho1—O1—K1	111.49 (12)
O4^vi^—Ho1—O4	67.63 (12)	K1^vi^—O1—K1	86.88 (9)
O4^vii^—Ho1—O4	126.77 (8)	W1—O2—K1^v^	132.6 (2)
O1—Ho1—O4	78.03 (12)	W1—O2—K1^ii^	115.89 (19)
O1^viii^—Ho1—O4	78.27 (12)	K1^v^—O2—K1^ii^	95.86 (13)
O3^ix^—Ho1—O4	143.86 (10)	W1—O2—K1^i^	99.64 (16)
O3^ii^—Ho1—O4	133.56 (10)	K1^v^—O2—K1^i^	120.18 (15)
O4^viii^—Ho1—O4	59.26 (13)	K1^ii^—O2—K1^i^	81.21 (11)
O3—K1—O2^v^	152.62 (12)	P1—O3—Ho1^xii^	96.20 (15)
O3—K1—O3^x^	78.69 (8)	P1—O3—K1	105.64 (14)
O2^v^—K1—O3^x^	124.78 (11)	Ho1^xii^—O3—K1	130.32 (12)
O3—K1—O1^vi^	119.65 (10)	P1—O3—K1^iii^	142.66 (16)
O2^v^—K1—O1^vi^	86.67 (12)	Ho1^xii^—O3—K1^iii^	95.30 (9)
O3^x^—K1—O1^vi^	60.35 (9)	K1—O3—K1^iii^	93.37 (11)
O3—K1—O2^xi^	93.55 (11)	P1—O4—Ho1^vi^	146.28 (18)
O2^v^—K1—O2^xi^	78.59 (14)	P1—O4—Ho1	99.72 (13)
O3^x^—K1—O2^xi^	80.53 (10)	Ho1^vi^—O4—Ho1	111.83 (11)
O1^vi^—K1—O2^xi^	118.34 (11)	P1—O4—K1	89.66 (13)
O3—K1—O4	52.05 (8)	Ho1^vi^—O4—K1	89.92 (10)
O2^v^—K1—O4	148.06 (12)	Ho1—O4—K1	110.96 (11)

Symmetry codes: (i) −*x*+1, −*y*+1/2, *z*; (ii) −*x*+3/2, *y*+1/2, *z*; (iii) *x*−1/2, −*y*, *z*; (iv) *x*−1/2, *y*, −*z*+1/2; (v) −*x*+3/2, −*y*+1/2, −*z*+1/2; (vi) −*x*+2, −*y*+1/2, *z*; (vii) *x*−1/2, −*y*+1/2, −*z*+1; (viii) −*x*+3/2, *y*, −*z*+1; (ix) *x*, *y*+1/2, −*z*+1; (x) *x*+1/2, −*y*, *z*; (xi) −*x*+3/2, *y*−1/2, *z*; (xii) *x*, *y*−1/2, −*z*+1.

## References

[bb1] Ben Amara, M. & Dabbabi, M. (1987). *Acta Cryst.* C**43**, 616–618.

[bb2] Blessing, R. H. (1995). *Acta Cryst.* A**51**, 33–38. 10.1107/s0108767394005726 7702794

[bb3] Brandenburg, K. (2006). *DIAMOND* Crystal Impact GbR, Bonn, Germany.

[bb4] Farrugia, L. J. (1999). *J. Appl. Cryst.* **32**, 837–838.

[bb5] Marsh, R. E. (1987). *Acta Cryst.* C**43**, 2470.

[bb6] Oxford Diffraction (2005). *CrysAlis CCD* and *CrysAlis RED* Oxford Diffraction Ltd, Abington, Oxfordshire, England.

[bb7] Sheldrick, G. M. (2008). *Acta Cryst.* A**64**, 112–122. 10.1107/S0108767307043930 18156677

[bb8] Zatovsky, I. V., Terebilenko, K. V., Slobodyanik, N. S. & Baumer, V. N. (2006). *J. Solid State Chem.* **179**, 3550–3555.

[bb9] Zatovsky, I. V., Terebilenko, K. V., Slobodyanik, N. S., Baumer, V. N. & Shishkin, O. V. (2006). *Acta Cryst.* E**62**, i193–i195. 10.1107/S160053680803287X PMC2959528 21580811

